# Associations between PON1 enzyme activities in human ovarian follicular fluid and serum specimens

**DOI:** 10.1371/journal.pone.0172193

**Published:** 2017-02-14

**Authors:** Keewan Kim, Michael S. Bloom, Victor Y. Fujimoto, Richard W. Browne

**Affiliations:** 1 Department of Environmental Health Sciences, University at Albany, State University of New York, Rensselaer, New York, United States of America; 2 Department of Epidemiology and Biostatistics, University at Albany, State University of New York, Rensselaer, New York, United States of America; 3 Department of Obstetrics, Gynecology, and Reproductive Sciences, University of California at San Francisco, San Francisco, California, United States of America; 4 Department of Biotechnical and Clinical Laboratory Sciences, University at Buffalo, State University of New York, Buffalo, New York, United States of America; Zhejiang University College of Life Sciences, CHINA

## Abstract

The importance of high-density lipoprotein (HDL) particle components to reproduction is increasingly recognized, including the constituent paraoxonase 1 (PON1). However, the reliability characteristics of PON1 enzymes in ovarian follicular fluid (FF) as biomarkers for clinical and epidemiologic studies have not been described. Therefore, we characterized PON1 enzymes in FF and serum and assessed the impact of the *PON1* Q192R polymorphism on associations between enzyme activities in two compartments. We also evaluated associations between HDL particle size and enzyme activities. We collected FF and serum from 171 women undergoing *in vitro* fertilization. PON1 activities were measured as paraoxonase and arylesterase activities, and HDL particle size was determined by ^1^H NMR spectrometry. Reliability indices for PON1 activities were characterized and we evaluated HDL particle sizes as predictors of PON1 enzyme activities. We found that PON1 enzyme activities were correlated between compartments, but higher in serum than in FF. For FF, the index of individuality (II) was low and the coefficient of variation (CV%) was high for paraoxonase activity overall (0.12 and 11.51%, respectively). However, IIs increased (0.33–1.30) and CV%s decreased (5.58%-8.52%) when stratified by *PON1* Q192R phenotype. The intraclass correlation coefficient (ICC) for FF paraoxonase activity was high overall (0.89) but decreased when stratified by *PON1* Q192R phenotype (0.43–0.75). We found similar, although more modest, patterns for FF arylesterase activity. For enzyme activities in serum, ICCs were close to 1.00 across all phenotypes. Additionally, different HDL particle sizes predicted PON1 enzyme activities according to *PON1* Q192R phenotype. Overall, stratification by *PON1* Q192R phenotype improved the reliability characteristics of FF PON1 enzymes as biomarkers for use in clinical investigations but diminished usefulness for epidemiologic studies. Thus, we recommend stratification by *PON1* Q192R phenotype for clinical but not epidemiologic investigations, when employing FF PON1 enzyme activity biomarkers.

## Introduction

High-density lipoprotein (HDL) provides the cholesterol substrate for steroid hormone synthesis in the human pre-ovulatory ovarian follicle [1], but also has well-recognized anti-oxidant and anti-inflammatory properties likely to be of importance for reproduction [2]. HDL functionality is driven, at least in part, by particle composition [3–5]. The HDL-particle comprises a dynamic micelle in which apolipoproteins frame a core of cholesteryl esters and triglycerides surrounded by a phospholipid monolayer; cholesterol, lipid-soluble micronutrients, and paraoxonase 1 (PON1) are integrated within [2]. HDL is the exclusive carrier of PON1, and circulating HDL particles cross the blood-follicle barrier and are the major source of PON1 in the ovarian follicle. Furthermore, PON1 is subject to several polymorphisms, the most studied of which is an arginine (R)-glutamine (Q) substitution at the 192^nd^ amino acid [3]. This *PON1* Q192R polymorphism is functional (meaning it affects enzyme activity) and the mutant R allele is particularly prevalent among Asians [6, 7]. Our group previously reported associations between the activity of follicular fluid (FF) PON1 and embryo quality among women undergoing *in vitro* fertilization (IVF) [8], presumably due to reduced lipid peroxidation [9].

The invasive nature of FF collection limits its application to IVF populations and there are limited data available to characterize associations with less invasively acquired serum specimens from non-clinical populations. With growing interest in the relevance of FF lipoproteins to human reproduction it is critical to assess suitability as biomarkers for use in clinical and epidemiologic applications [10]. To address the pending data gap, we augmented our recent characterization of distributions, sources of variability and reliability characteristics for FF HDL-particle constituents in addition to those measured in serum among 171 IVF patients [11–13]. Here we characterized *PON1* enzymes in FF and serum measured as paraoxonase and arylesterase activities, and assessed the impact of the *PON1* Q192R polymorphism on associations between *PON1* enzymes in FF and serum. Given that the anti-oxidant activity of HDL is, in part, determined by particle size, we further assessed associations between HDL particle size and enzyme activities according to the *PON1* Q192R polymorphism.

## Materials and methods

### Sample selection

Our study was conducted within the usual clinical context of IVF procedures at the University of California at San Francisco (UCSF) Center for Reproductive Health (USA). Sample recruitment and the clinical study protocol were previously described in detail [11]. Briefly, we enrolled a convenience sample of 180 women undergoing IVF treatment at UCSF from April 10^th^, 2010 to June 28^th^, 2011. Following controlled gonadotropin-induced ovarian follicle stimulation (COS) according to clinic protocols and subcutaneous administration of human chorionic gonadotropin (hCG), oocytes were retrieved using transvaginal fine needle aspiration and blood specimens were obtained on the same day. We employed a single-follicle design in which the largest follicle (>17 mm diameter) present on each contralateral ovary was aspirated individually and separately from the remaining follicle cohort. Residual, undiluted FF from two separate contralateral follicles and blood specimens were retained and processed for analysis (n = 171) by the Clinical Biochemistry and Oxidative Stress Laboratory at the University of Buffalo, State University of New York (USA). A separate aliquot was sent to LipoScience, Inc. (Chicago, Illinois, USA) for HDL particle size quantification. All participants provided written informed consent prior to study enrollment and the study protocol was approved by the UCSF Committee on Human Research.

### Biochemical analysis

PON1 arylesterase and paraoxonase activities in FF and serum, and the *PON1* Q192R polymorphism phenotype, were determined in duplicate as previously described [14]. In brief, paraoxonase activity (IU/L) was determined by the rate of formation of p-nitrophenol at 412 nm using 1 mmol/L paraoxon as the substrate in 50 mmol/L glycine buffer, pH 10.5, with 1.0 mmol/L CaCl_2_ with and without 1 mol/L NaCl. Arylesterase activity (kIU/L) was determined by the rate of formation of phenol at 270 nm using 4 mmol/L phenyl acetate as the substrate in 20 mM Tris-HCl, pH 8.0, with 1.0 mM CaCl_2_. Arylesterase activity with p-nitrophenyl acetate as substrate was determined only for use in phenotype assignment and was determined as the rate of formation of p-nitrophenol at 405 nm in 25 mmol/L TEA buffer, pH 7.4, with 1.0 mmol/L CaCl_2_ with (inhibited arylesterase activity (IA)) or without (non-inhibited arylesterase activity (NIA)) 1 mmol/L phenyl acetate. Water blanks were used to correct for non-enzymatic hydrolysis. The coefficients of variation (CV) were 0.6%-1.4% for *PON1* assays. PON1 phenotype was assigned based on the ratio of PON1 enzyme activities using these different analytical conditions and substrates. This activity ratio phenotype has been proven to be 100% accurate in assigning *PON1* Q192R phenotype in comparison to Alw1 restriction fragment length polymorphism (i.e., genotype determination) [14].

We used proton nuclear magnetic resonance spectrometry (^1^H NMR) to classify HDL particles in serum and FF as large (9.7–13.5 nm), medium (8.3–9.4 nm), and small (7.4–8.2 nm) sizes according to diameter (LipoScience, Inc.) We further quantified size-specific FF HDL particles by identifying NMR signals unique to specific HDL particle sizes according to a previously described method [15]. We obtained concentrations of 26 HDL particle sizes in FF, ranging from 7.4 nm to 13.5 nm diameter.

### Statistical analysis

We normalized PON1 enzyme activities and concentrations of HDL particles and stabilized variances using a natural log transformation prior to data analysis. The distribution of *PON1* Q192R phenotypes was characterized by demographic and clinical factors using the χ^2^-test or ANOVA as appropriate. To assess similarities between biologic compartments, we compared PON1 enzyme activities in FF and serum by paired Student-T tests in n = 141 with available data. We also evaluated linear associations between compartments using Pearson correlations. Among n = 118 with no missing values (i.e., balanced data set), we employed two-stage nested ANOVA to characterize sources of variability between-women $\left(\sigma_{B}^{2}\right)$ and between-follicles $\left(\sigma_{F}^{2}\right)$, and due to analytic factors $\left(\sigma_{A}^{2}\right)$, which included random variation, laboratory variability, and other factors not captured by $\sigma_{B}^{2}$ or $\sigma_{F}^{2}$. The relative contribution of each variability source to total measurement variability was calculated and we used overlap of 84% confidence intervals (CI) to evaluate significant differences in geometric mean values [16]. We characterized the index of individuality (II), to assess the utility of population reference ranges in clinical settings [17]. We also calculated coefficients of variation (CV%) and intraclass correlation coefficients (ICC), with 95% CIs estimated using the inverse tan transformation of Smith’s variance [18]. Finally, we determined ‘k,’ the minimum number of specimens required to estimate the woman-specific mean value with 10% error [17].

For multivariable analysis, we used linear regression models with HDL particles as predictors and PON1 enzyme activities as the outcomes, stratified by *PON1* Q192R phenotype. Large, medium, and small size FF and plasma HDL particle groups were simultaneously entered into regression models as independent variables. For HDL particles and enzyme activities measured in FF, we incorporated generalized estimating equations (GEE) to account for the correlated nature of FF measures made within woman [19]. All models were adjusted for *a priori* identified confounders, including age in years [20], body mass index (BMI) in kg/m^2^ [21, 22], and cigarette smoking as ‘never’ vs. ‘ever’ [23]. We further assessed 26 size-specific FF HDL particles as predictors of FF PON1 enzyme activities using a forward stepwise selection procedure. Briefly, we screened 26 HDL particle sizes by regressing each on PON1 enzyme activities stratified by *PON1* Q192R phenotype and retained only those HDL particles with P < 0.05. We employed a change in quasi-likelihood criterion (QIC) of 1.00 to retain or remove FF HDL particle sizes from the final regression models. Exponentiated regression coefficients from the final models are presented as % change in enzyme activities per ln-transformed μmol/L FF HDL and 95% CIs. SAS v.9.3 (SAS Institute, Cary, NC USA) was used for the analysis and statistical significance was defined as P < 0.05 for a two-tailed test.

## Results

### PON1 enzyme activities were higher in serum than in FF, irrespective of Q192R phenotype

**Fig 1:**
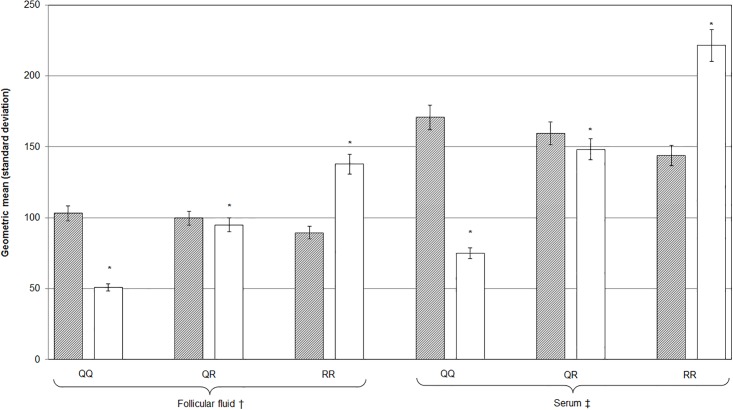
Distribution of PON1 enzyme activities measured in FF and serum, stratified by *PON1* Q192R phenotype. Geometric means and standard deviations (SD) of arylesterase activity (shaded bars) and paraoxonase activity (open bars) presented; differences for enzyme activities in FF and serum are significant (P < 0.0001) within each *PON1* Q192R phenotype; * P < 0.0001 for difference across *PON1* Q192R phenotypes; ^†^ n = 10 missing values; ^‡^ n = 14 missing values.

As described by Table 1 most participants were assigned either the QQ (40.9%) or QR (42.6%) *PON1* Q192R phenotype; 16.4% presented with the homozygous RR *PON1* Q192R polymorphism. *PON1* Q192R phenotype differed by race (P < 0.0001). The QQ polymorphism was less prevalent among Asians (14.9%) than among non-Asians (48.7%). In contrast, the RR polymorphism was common among Asians (31.9%) but infrequent among non-Asians (10.4%). The QQ phenotype conferred higher arylesterase and lower paraoxonase activities, whereas those with the RR phenotype had lower arylesterase and higher paraoxonase activities; the difference was statistically significant only for paraoxonase activity (Fig 1). We detected no differences by age, BMI, cigarette smoking, diagnosis, or COS protocol.

**Table 1:** Distribution of demographic and clinical factors by *PON1* Q192R phenotype among *in vitro* fertilization patients (n = 171).

Factor	*PON1* Q192R phenotype					
	QQ (n = 70)		QR (n = 73)		RR (n = 28)	
	n	%	n	%	n	%
Age (years)						
< 35 years old	26	54.2	18	37.5	4	8.3
≥ 35 years old	44	35.8	55	44.7	24	19.5
BMI (kg/m^2^)						
< 25	51	46.4	42	38.2	17	15.5
25–29	13	31.0	22	52.4	7	16.7
≥ 30	6	31.6	9	47.4	4	21.1
Race ^a^^,^ *						
non-Asian	56	48.7	47	40.9	12	10.4
Asian	7	14.9	25	53.2	15	31.9
Cigarette smoking ^b^						
Never	56	38.4	64	43.8	26	17.8
Ever	9	47.4	8	42.1	2	10.5
Diagnosis						
Male factor	28	45.2	26	41.9	8	12.9
Unexplained ^c^	18	37.5	18	37.5	12	25.0
Female factor-non DOR ^d^	13	43.3	14	46.7	3	10.0
Female factor-DOR	9	33.3	14	51.9	4	14.8
PGD-only	2	50.0	1	25.0	1	25.0
COS protocol						
Lupron down regulated	48	41.7	48	41.7	19	16.5
Antagonist	18	42.9	17	40.5	7	16.7
Flare	4	28.6	8	57.1	2	14.3

^a^ n = 9 missing values;

^b^ n = 6 missing values;

^c^ includes n = 1 recurrent pregnancy loss;

^d^ includes n = 10 endometriosis, n = 10 tubal factor, n = 7 polycystic ovary syndrome and n = 3 anovulation;

* P < 0.05 for across *PON1* Q192R phenotypes.

BMI, body mass index; COS, controlled ovarian stimulation; DOR, diminished ovarian reserve; FF, follicular fluid; PGD, pre-implantation genetic diagnosis.

For all phenotypes, enzyme activities were significantly (P < 0.0001) higher in serum than in FF (Fig 1). Absolute differences in FF and serum arylesterase activities decreased with an increasing number of R alleles (i.e., QQ = 67.6, QR = 59.6, RR = 54.3 kIU/L), whereas paraoxonase activity differences increased with more R alleles (i.e., QQ = 23.9, QR = 53.2, RR = 83.7 IU/L). Still, we detected strong and moderate correlations between enzyme activities measured in FF and serum for paraoxonase (r = 0.80, P < 0.0001) and arylesterase (r = 0.46, P < 0.0001).

### CVs were low, with ICCs low for FF and high for serum PON1 enzyme activities

In Table 2, sources of variability and reliability indices are described for arylesterase and paraoxonase activities overall and according to *PON1* Q192R phenotype. For PON1 activities measured in FF, variability between women was the major contributor to each, with the exception of paraoxonase activity among women with the RR phenotype. For the latter, sources between-follicles was the main contributor (56.04%) to overall variability and this value was significantly higher than for the QR (23.08%) or QQ (21.58%) phenotypes. Variability attributed to analytic factors was uniformly low, although the contribution to paraoxonase was significantly higher in the QQ (3.16%) phenotype than in the QR (1.24%) and RR phenotypes (0.56%). II values were low for arylesterase activity overall (0.26), and similar when stratified by phenotype (0.23–0.30). Although II was also low for overall paraoxonase activity (0.12), values were substantially increased when stratified by phenotype (0.32–1.30). A single specimen collection was sufficient to characterize woman-specific mean values in all scenarios. CVs were mostly below 10%, although 11.51% for paraoxonase activity overall. ICC values approximated or exceeded 0.80 (0.75–0.89) for all groups with the exception of RR phenotype paraoxonase activity (0.43). Variability attributed to analytic factors for serum arylesterase activity was higher for QQ (1.62%) than for QR (0.90%) and RR (0.94%) phenotypes, whereas serum paraoxonase variability attributed to analytic factors was significantly different across all phenotypes. For PON1 activities measured in serum, CVs were below mostly 10%, similar to those measured in FF, and ICC values were close to 1.00 across all *PON1* Q192R phenotypes.

**Table 2:** Characteristics of biological variability for concentrations of PON activities measured in follicular fluid (FF) and serum by *PON1* Q192R phenotype among *in vitro* fertilization patients.

PON Q192R Phenotype	n	σ^2^_T_	%σ^2^_B_	%σ^2^_F_	%σ^2^_A_	II	k	CV%	ICC	95% CI	
FF											
Overall											
Arylesterase (kIU/L)	118	0.132	79.29	19.28	1.44	0.26	1	7.90	0.79	0.75	0.84
Paraoxonase (IU/L)	118	0.254	89.45	9.80	0.75	0.12	1	11.51	0.89	0.87	0.92
QQ											
Arylesterase (kIU/L)	48	0.122	81.43	16.80	1.77 ^a^	0.23	1	7.58	0.81	0.75	0.88
Paraoxonase (IU/L)	48	0.111	75.26	21.58 ^a^	3.16 ^a^	0.33	1	8.52	0.75	0.67	0.84
QR											
Arylesterase (kIU/L)	48	0.121	77.12	21.48	1.39 ^a^^,^ ^b^	0.30	1	7.46	0.77	0.69	0.85
Paraoxonase (IU/L)	48	0.076	75.68	23.08 ^a^	1.24 ^b^	0.32	1	6.02	0.76	0.67	0.84
RR											
Arylesterase (kIU/L)	22	0.175	78.71	20.25	1.04 ^b^	0.27	1	9.31	0.79	0.67	0.90
Paraoxonase (IU/L)	22	0.076	43.40	56.04 ^b^	0.56 ^c^	1.30	1	5.58	0.43	0.19	0.68
Serum											
Overall											
Arylesterase (kIU/L)	157	0.092	98.91	−	1.09	−	1	5.95	0.99	0.99	0.99
Paraoxonase (IU/L)	157	0.282	99.84	−	0.16	−	1	11.08	1.00	1.00	1.00
QQ											
Arylesterase (kIU/L)	62	0.065	98.38	−	1.62 ^a^	−	1	4.95	0.98	0.98	0.99
Paraoxonase (IU/L)	62	0.093	99.14	−	0.86 ^a^	−	1	7.06	0.99	0.99	1.00
QR											
Arylesterase (kIU/L)	70	0.093	99.10	−	0.90 ^b^	−	1	6.02	0.99	0.99	1.00
Paraoxonase (IU/L)	70	0.140	99.79	−	0.21 ^b^	−	1	7.47	1.00	1.00	1.00
RR											
Arylesterase (kIU/L)	25	0.139	99.06	−	0.94 ^b^	−	1	7.52	0.99	0.98	1.00
Paraoxonase (IU/L)	25	0.097	99.89	−	0.11 ^c^	−	1	5.78	1.00	1.00	1.00

NOTE: All values were natural log-transformed for the analyses and only women with no missing values were used (i.e., balanced data).

^a, b, c^ Different superscript letters indicate P < 0.05 for difference in $\%\sigma_{F}^{2}$ and $\%\sigma_{A}^{2}$ values between phenotypes for arylesterase or paraoxonase activities. For example, $\%\sigma_{F}^{2}$ in FF paraoxonase activity for RR phenotype is significantly different (P < 0.05) from QQ and QR phenotypes.

CI, confidence interval; CV, coefficient of variation; ICC, intraclass correlation coefficient; II, index of individuality; k, minimum number of specimens required to estimate the women-specific mean value within 10% of the true value; $\sigma_{A}^{2}$, variability attributed to analytic factors; $\sigma_{B}^{2}$, variability between-women; $\sigma_{F}^{2}$, variability between-follicles; $\sigma_{T}^{2}$, total variability.

### PON1 enzyme activities were predicted by large, medium, and small HDL particles in FF, although HDL particle predictors in plasma varied

As presented in Table 3, FF HDL particles were associated with FF PON1 enzyme activities regardless of HDL particle size group (e.g., large, medium, and small) or *PON1* Q192R phenotype. On the other hand, only medium and small size HDL particles measured in plasma predicted serum arylesterase activity for the QR phenotype, with no impact for the QQ or RR phenotypes. For serum paraoxonase activity, plasma HDL particle predictors varied by phenotype.

**Table 3:** Multivariable linear regression models of follicular fluid (FF) and serum PON1 activities associated with high-density lipoprotein (HDL) particles (μmol/L), according to *PON1* Q192R phenotype.

PON activity	Phenotype	HDL particles	FF ^a^			Serum		
			% change	95% CI		% change	95% CI	
				Low	High		Low	High
Arylesterase activity	QQ	Large	1.22 *	1.14	1.31	1.11	0.88	1.40
		Medium	1.34 *	1.21	1.49	1.14	0.98	1.31
		Small	1.64 *	1.46	1.84	0.99	0.89	1.10
	QR	Large	1.12 *	1.01	1.25	1.25	0.87	1.81
		Medium	1.40 *	1.19	1.64	1.24 *	1.02	1.52
		Small	1.92 *	1.65	2.24	1.24 *	1.07	1.45
	RR	Large	1.20 *	1.06	1.35	1.11	0.67	1.83
		Medium	1.43 *	1.31	1.56	1.00	0.63	1.58
		Small	1.55 *	1.33	1.80	1.30	0.81	2.10
Paraoxonase activity	QQ	Large	1.13 *	1.04	1.22	1.43 *	1.12	1.81
		Medium	1.32 *	1.20	1.46	1.21 *	1.04	1.41
		Small	1.70 *	1.53	1.90	1.04	0.93	1.17
	QR	Large	1.11 *	1.04	1.19	1.41	0.89	2.24
		Medium	1.33 *	1.23	1.44	1.32 *	1.02	1.70
		Small	1.74 *	1.59	1.91	1.27 *	1.05	1.54
	RR	Large	1.24 *	1.14	1.36	1.80 *	1.16	2.77
		Medium	1.58 *	1.46	1.69	1.25	0.83	1.86
		Small	1.57 *	1.37	1.80	1.19	0.78	1.79

NOTE: PON enzyme activities and concentrations of HDL particles were natural log-transformed for the analyses. Models were adjusted for age, body mass index, and cigarette smoking.

^a^ Generalized estimating equations were used to provide robust standard errors;

* indicates P < 0.05; CI, confidence interval.

Using 27 size-specific HDL particles measured in FF, we identified that different individual HDL particles sizes predicted different PON1 enzyme activities according to *PON1* Q192R phenotypes (Table 4). For the QQ and QR phenotypes, the 8.0–9.4 nm medium-sized FF HDL particles were significant predictors of FF arylesterase activity. On the other hand, for the RR phenotype only the 13.0 nm large-sized HDL particles significantly predicted FF arylesterase activity (21.39% change, 95% CI 2.52–181.62), although this effect estimate was imprecise due to small sample size. Across *PON1* Q192R phenotypes, we identified various HDL particle sizes of 8.0 to 9.7 nm diameter as significant, confounder-adjusted predictors of FF paraoxonase activity.

**Table 4:** Multivariable linear regression models of follicular fluid (FF) PON1 activities associated with size-specific high-density lipoprotein (HDL) particles (μmol/L), according to *PON1* Q192R phenotype.

PON activity	Phenotype	HDL particle size (nm)	n women (n follicles)	% change	95% CI	
					Low	High
Arylesterase activity	QQ	9.4	59 (102)	1.25 *	1.07	1.46
	QR	8.3	63 (110)	0.74 *	0.62	0.87
	-	8.0	-	1.10 *	1.03	1.16
	RR	13.0	28 (50)	21.39 *	2.52	181.62
Paraoxonase activity	QQ	9.7	59 (102)	1.23 *	1.09	1.40
	QR	8.0	63 (110)	1.14 *	1.06	1.21
	RR	8.5	28 (50)	1.32 *	1.14	1.52

NOTE: PON activities and concentrations of specific HDL particle sizes were natural log-transformed for the analyses. Models were adjusted for age, body mass index, and cigarette smoking, and generalized estimating equations were used to provide for robust standard errors.

* indicates P < 0.05;

CI, confidence interval.

## Discussion

Here we describe the distribution of PON1 enzyme activities in FF and serum by *PON1* Q192R phenotype, calculate reliability indices, and identify *PON1* Q192R phenotype-specific HDL-particle sizes associated with enzyme activities in a cohort of women undergoing IVF. The variability of PON1 enzyme activities in serum have been characterized before [24], and levels were previously described by *PON1* Q192R phenotype [14]. Yet, these are the first data to our knowledge to describe variability sources in human FF by PON1 phenotype and to characterize their utility as biomarkers for clinical and epidemiologic studies. Furthermore, we identified specific HDL particle sizes as predictors of PON1 enzyme activities according to *PON1* Q192R phenotype.

Not surprisingly, the *PON1* Q192R phenotype R allele was more prevalent in Asian participants than in non-Asian participants [6, 7]. The R allele confers a higher rate of paraoxonase activity than the Q allele [25], and in fact our team [11] and others [26] previously reported significantly higher paraoxonase activity in Asians compared to other groups. Paraoxonase activity is believed to account in large part for the antioxidant activities of HDL [27, 28]; higher activity enhances defense against reactive oxygen species [29, 30] and thereby may confer a reproductive advantage [31, 32]. We detected no significant difference in the distribution of PON1 phenotype by clinical factors, including infertility diagnosis, although a lower proportion of women diagnosed as unexplained infertility were assigned the RR phenotype.

Despite lower PON1 enzyme activities in FF compared to serum for all PON1 phenotypes, enzyme activities measured in both compartments were significantly inter-correlated. Considering that the source of FF HDL particles is derived from serum through the blood-follicle barrier our results may suggest serum to be an equivalent biomarker for FF PON1 enzyme activities, and is certainly less invasive. However, the correlation between FF and serum compartments varied substantially for enzyme substrates, in which paraoxonase activities were more strongly correlated (r = 0.80) than arylesterase activities (r = 0.46). This may limit the use of serum as a biomarker for overall FF PON1 enzyme activities, although potentially useful to characterize paraoxonase activity. Still, replication of these results using a larger sample size will be required for a more definitive interpretation.

We attributed a majority of total variability in FF PON1 enzyme activities to sources between-women, although sources between-follicles were highest for the RR phenotype. Overall II values were consistent with those reported for arylesterase (0.32) and paraoxonase (0.24) activities in serum collected from nine normally cycling women 31–45 years of age in a previous study [24], and with values of 0.31 and 0.19, respectively, reported for 17 women and men from a Western New York State population-based control group [14]. From a clinical perspective, low II indicates limited dispersion of individual measurements across the distribution of measurements between women, and so an abnormal value is unlikely to breach population reference intervals [33]. However, paraoxonase activity II increased when stratifying according to *PON1* Q192R phenotype; from 0.12 to 1.30 for women assigned as RR, approaching the 1.4 threshold promulgated for suitability [33]. Likewise, the CV exceeded 10% for overall paraoxonase activity, a threshold below which random error contributes not more than 10% to the mean estimate, suggesting suitability of a biomarker for clinical use [34, 35]. However, all phenotype-specific paraoxonase activity CVs declined to less than 10% (5.58%-8.52%). Overall and for all phenotypes, a single FF collection was sufficient to characterize the subject specific mean enzyme activities within 10% error, consistent with serum PON1 in our data and with previous observations [14, 24]. Generally, *PON1* Q192R phenotype stratification improved the reliability characteristics of FF PON1 enzymes for clinical use, in agreement with previous studies indicating the need to simultaneously consider activity and phenotype for characterizing PON1 status [36, 37].

The ICC describes the proportion of total measurement variability attributed to sources between-women, or alternately the correlation of replicate measures between-follicles. In contrast to the CV, which evaluates reliability at the mean of measured values, the ICC evaluates reliability across the range of measured values and is thus of greater consequence for population level studies [38]. To maintain statistical power for detecting differences, ICC values below 0.8 necessitate sample size increases of at least 25% and also reduce criterion validity, thus establishing a useful suitability threshold for employment in epidemiologic studies [18]. We identified ICC values with 95% CIs overlapping 0.80 for PON1 enzyme activities, although modestly lower than reported for arylesterase (0.98, 95% CI 0.95–0.98) and paraoxonase (0.99, 95% CI 0.98–0.99) using serum from the aforementioned sample of nine normally cycling women [24]. The latter is likely a consequence of the higher variability between-follicles for arylesterase and paraoxonase activities in our study compared to the longitudinal serum variability within-person in the prior study (1.20% and 1.40%, respectively) [24]. However, we also identified lower ICCs when stratifying by *PON1* Q192R phenotype, in particular for RR paraoxonase activity. Generally, *PON1* Q192R phenotype stratification may not be an efficient strategy for epidemiologic studies using FF PON1 as a biomarker, in particular among Asian populations, for whom the RR phenotype has high prevalence. Similarly, as evidenced by ICCs close to 1.00 across *PON1* Q192R phenotypes for both arylesterase and PON1 activities, stratification did not improve the reliability characteristics for serum PON1 activities, although the values suggest that serum is an appropriate biomarker for epidemiologic investigations.

As the anti-oxidant activity of HDL is, in part, determined both by particle size and PON1, which is integrated within the structure [2], we investigated whether specific HDL particle size predicted enzyme activities by *PON1* Q192R phenotype. Indeed, particular plasma HDL size was associated with different serum PON1 enzyme activity levels according to phenotype in our data. Yet, we did not identify a common HDL particle size pattern predictive of PON1 enzyme activities across phenotypes. In FF, we detected that all HDL particles measured for large, medium, and small size groups were associated with PON1 activities. Using FF HDL measured for 26 different size particles, we found that specific HDL particle size predicted enzyme activities by *PON1* Q192R phenotype. Though such detailed data for plasma were unavailable to us, overall our data suggest that the *PON1* Q192R phenotype plays a more important role in governing PON1 enzyme activities than that played by HDL particle size. An explanation regarding the differences observed in activity and particle size with PON phenotype is not clear. However, it is clear that PON1 is present across the HDL particle range but is found preferentially in the smaller and more dense HDL3 subclass (relative to the larger HDL2 subclass) [39, 40]. The Q192R polymorphism, in contrast to the L55M and the T(−107)C polymorphisms, had an effect on the distribution profile of PON1 activity in previous research [41]. Indeed, serum PON1 Q192R polymorphs differ in HDL binding, lipolactonase stimulation, and cholesterol efflux capacity [42]. In our data, the association between PON activity, genotype, and particle size was generally stronger in FF than in serum. This indicates that these associations are further dependent on additional factors that variously impact Q192R alleles and may not all be present in FF, but are measured in serum, including the presence of other lipoproteins, the basal level of oxidative stress and lipid peroxidation products [8–10], or other xenobiotic environmental substances [43]. Still, our analysis was limited by small sample size, particularly for paraoxonase activity by RR phenotype, which led to imprecise effect estimates. Future examination of FF paraoxonase activities should incorporate measures of these pleiotropic effectors in a more comprehensive study design, and using a larger sample.

## Conclusions

In conclusion, we identified similar reliability characteristics for FF PON1 enzyme activities as serum measures. Stratification by the *PON1* Q192R phenotype improved biomarker characteristics for FF PON1 enzyme activities in terms of their likely performance in clinical settings, yet appeared to diminish their suitability for use in population-level epidemiologic studies. Additional experiments about the underlying mechanism are necessary to elucidate reasons why PON1 Q192R turned out to be suitable for clinical investigations. In contrast, serum PON1 enzyme activities appeared suitable for population-level investigations. Although all FF HDL particles determined for large, medium, and small size were positively associated with PON1 activities, specific particle sizes may be important factors for predicting enzyme activities. Still, it is important to recognize the limitations of our results given the promiscuity of the PON1 enzyme substrates employed [42, 44]. Given the increasing interest in FF constituents as biomarkers in clinical and research settings and a strong likelihood for PON1 to impact reproductive outcomes, these data should prove useful in guiding clinical use and in designing epidemiologic studies of oxidative stress and IVF endpoints.

## Supporting information

S1 Dataset Study data. (XLSX)
